# Measuring Proviral HIV-1 DNA: Hurdles and Improvements to an Assay Monitoring Integration Events Utilising Human *Alu* Repeat Sequences

**DOI:** 10.3390/life11121410

**Published:** 2021-12-16

**Authors:** Eva Malatinkova, Jordan Thomas, Ward De Spiegelaere, Sofie Rutsaert, Anna Maria Geretti, Georgios Pollakis, William A. Paxton, Linos Vandekerckhove, Alessandra Ruggiero

**Affiliations:** 1 HIV Cure Research Center, Department of Internal Medicine, Faculty of Medicine and Health Sciences, Ghent University, B-9000 Ghent, Belgium; e.malatinkova@gmail.com (E.M.); Sofie.Rutsaert@UGent.be (S.R.); Linos.Vandekerckhove@UGent.be (L.V.); 2 Department of Clinical Infection, Microbiology and Immunology, Institute of Infection, Veterinary and Ecological Sciences, University of Liverpool, Liverpool L69 7BE, UK; hljthom2@student.liverpool.ac.uk (J.T.); G.Pollakis@liverpool.ac.uk (G.P.); W.A.Paxton@liverpool.ac.uk (W.A.P.); 3 Laboratory of Veterinary Morphology, Faculty of Veterinary Sciences, Ghent University, B-9820 Ghent, Belgium; Ward.despiegelaere@ugent.be; 4 Fondazione PTV and Faculty of Medicine, University of Rome Tor Vergata, 00133 Rome, Italy; anna_maria.geretti@kcl.ac.uk; 5 School of Immunology & Microbial Sciences, King’s College London, London WC2R 2LS, UK; 6 Department Neurosciences, Biomedicine and Movement Sciences, School of Medicine-University of Verona, 37129 Verona, Italy

**Keywords:** integrated HIV-1, *Alu*-gag, *Alu*-5LTR, quantification, reservoir

## Abstract

Integrated HIV-1 DNA persists despite antiretroviral therapy and can fuel viral rebound following treatment interruption. Hence, methods to specifically measure the integrated HIV-1 DNA portion only are important to monitor the reservoir in eradication trials. Here, we provide an up-to-date overview of the literature on the different approaches used to measure integrated HIV-1 DNA. Further, we propose an implemented standard-curve free assay to quantify integrated HIV-1 DNA, so-called *Alu*-5LTR PCR, which utilises novel primer combinations. We tested the *Alu*-5LTR PCR in 20 individuals on suppressive ART for a median of nine years; the results were compared to those produced with the standard-free *Alu*-gag assay. The numbers of median integrated HIV-1 DNA copies were 5 (range: 1–12) and 14 (5–26) with the *Alu*-gag and *Alu*-5LTR, respectively. The ratios between *Alu*-gag vs *Alu*-5LTR results were distributed within the cohort as follows: most patients (12/20, 60%) provided ratios between 2–5, with 3/20 (15%) and 5/20 (25%) being below or above this range, respectively. *Alu*-5LTR assay sensitivity was also determined using an “integrated standard”; the data confirmed the increased sensitivity of the assay, i.e., equal to 0.25 proviruses in 10,000 genomes. This work represents an improvement in the field of measuring proviral HIV-1 DNA that could be employed in future HIV-1 persistence and eradication studies.

## 1. Introduction

Current antiretroviral therapy (ART) is ineffective at eradicating human immunodeficiency virus type-1 (HIV-1) infection, as the virus stably integrates its DNA into the genome of long-lived target cells, which include CD4 T cells, monocytes, and macrophages [1,2,3,4,5,6]. Specific T cell subsets are thought to be key hosts of the HIV-1 latent reservoir, and therefore, important contributors to viral persistence. For example, follicular T cells (T_fh_) within the B cell follicle have recently been shown to be enriched in replication competent provirus [7,8,9,10,11]. Similarly, a high proportion of regulatory T cells (Treg) and Th17 cells has been shown to harbour HIV-1 provirus [12,13,14,15,16]. Recently, CD32+ T cells have been identified as a reservoir of proviral DNA [17,18]; however, the contribution of these cells to the viral reservoir remains controversial [19,20,21,22]. Despite the vast majority of integrated HIV-1 DNA sequences being replication deficient due to mutations introduced during reverse transcription [23,24,25,26], the small proportion of provirus that retains replication competence is sufficient to facilitate virus rebound within weeks of ART cessation [27,28,29]. The latent reservoir (LR) is therefore considered the primary barrier to HIV-1 eradication and must be specifically and accurately measured in order to assess the efficacy of novel cure strategies [6].

Cell-based assays, such as the quantitative viral outgrowth assay (QVOA), measure the production of HIV-1 from stimulated CD4 T cells, and are considered the gold standard method for LR quantitation due to their ability to measure only replication-competent provirus [20,30,31,32,33,34,35]. The first generation QVOA assays are known to underestimate the size of the replication competent reservoir by excluding intact proviruses that require multiple rounds of stimulation for viral outgrowth [24]. Additionally, they are laborious and time consuming, requiring large amounts of patient material [25]. Next generation QVOAs aim to reduce sample material and the time to assay read out [36,37]. Nevertheless, a recent analysis of QVOA revealed significant variation in sample quantification from multiple labs, suggesting that meaningful comparisons of results from clinical studies may be limited [38]. Alternative methods to quantify intracellular HIV-1 reservoirs have been developed with the view to improve practicality whilst maintaining accuracy. One explored possibility is the measurement of cell-associated RNA as a surrogate of virus replication following cell stimulation; however, this does not indicate the replication competency of the virus [39,40,41].

With improvements in sequencing techniques, some have proposed the use of next generation sequencing (NGS) to measure the proviral reservoir [42,43,44,45,46,47]. NGS can be used both to characterise viral sequences and define integration sites, that can be used to track the clonal expansion and persistence of infected cells, as well as integration site data, which can be used to identify proviruses that affect the growth and survival of the infected cells [48]. Methods such as Full-Length Individual Proviral Sequencing (FLIPS) have been developed to identify functional proviruses [42], but without identifying the provirus integration site [49]. As described in Gao H et al. [47], the ligase-mediated (LM) PCR [50], linear-amplification-mediated (LAM) PCR [51], linker-mediated PCR [52], inverse PCR [53], and, most recently, nonrestrictive (nr) LAM PCR [54,55] can be used to characterise HIV-1 provirus integration sites, but these approaches do not provide full genome sequences. More recently, matched integration site and proviral sequencing (MIP-Seq) and multiple-displacement amplification-single-genome sequencing (MDA-SGS) have made it possible to sequence the individual proviruses to be linked to the integration site in the genome [43,49,56]. Multiple displacement amplification (MDA) facilitates amplification of the whole genome from patient samples, producing 1000–10,000 identical copies of an individual cell’s genome, including any proviral sequence that it may harbour [43]. As described in Einkauf et al., this has been combined with viral genome sequencing using primers spanning near-full-length HIV-1 [5,57], and with chromosomal integration site analysis based on integration site loop amplification (ISLA) [58], ligation-mediated PCR (LM-PCR) [59], or nonrestrictive linear amplification-mediated PCR (nrLAM-PCR) [55]. Results generated from MDA have highlighted that prolonged ART enriches for intact integrated HIV-1 genomes into nongenic regions or in the reverse orientation to the host gene, enforcing deep viral latency [43,56]. This year, Artesi M. et al. presented a novel approach called Pooled CRISPR Inverse PCR sequencing (PCIP-seq), which allows for the simultaneous identification of the integration site and tracking of clone abundance while also sequencing the provirus inserted at that position [49]. Nevertheless, the feasibility of use of NGS-based assays in clinical settings may be limited due to large sample volumes and costs.

A widely used alternative is the measurement of intracellular HIV-1 DNA using PCR based assays, even though they do not distinguish defective proviruses [4,6]. Total HIV-1 DNA in infected cells can be found in two forms: integrated HIV-1 DNA (provirus) and unintegrated HIV-1 DNA, which includes linear or dead-end circular episomal DNA that recombine to form 1-or 2-LTR circles [6,53,60]. Total HIV-1 DNA quantification assays are high-throughput and relatively inexpensive; however, substantial variation exists when comparing different assays due to the diversity of the HIV-1 genome and consequent primer/target mismatches [61,62]. Unlike total HIV-1 DNA quantification methods, measurements of proviral HIV-1 DNA can be more challenging, because they rely on the amplification of the junction between human genome and HIV using an HIV-1 specific primer and a primer that anneals to human *Alu* sequences that are interspersed within the human genome at about 5 kb distance from each other [4,52,63,64,65,66,67]. One rigorous study by Erikson [68] compared all the methods used to measure the HIV-1 reservoir, showing that total and integrated HIV-1 DNA quantification assays correlate significantly in ART suppressed patients, likely due to the paucity of unintegrated DNA forms in the absence of ongoing replication [68]. This analysis also demonstrated that measuring integrated HIV-1 DNA using the so-called *Alu*-gag PCR correlated well with results obtained from QVOA, and together, these findings suggest that integrated HIV-1 DNA quantification may be the most appropriate PCR-based method for LR measurement [68].

One hurdle in measuring integrated HIV-1 is represented by the natural heterogeneity of HIV-1 integration which results in poor PCR efficiency. As such, if using a quantification standard to measure integrations, this should contain multiple integration sites [64]. To obviate to this, we recently optimised the *Alu*-gag assay for standard-free quantification of integrated HIV-1 DNA in peripheral blood mononuclear cells (PBMC) using Poisson statistics [4,65,67]; of note, this assay was demonstrated to correlate well with QVOA, thus, providing some indications on the replication competency of the virus [69]. Another hurdle is the linear amplification of nonintegrated DNA forms, though this issue has been addressed by simultaneous amplification with only the HIV-1 primer to distinguish them from integrated provirus [70,71]. Because of these limitations, the *Alu*-gag HIV-1 PCR assay has been demonstrated to detect 10% of integration events, translating to 0.5 proviruses in 10,000 genomes [65,71]. This limit of detection is a reflection of the efficiency of PCR which is determined by different factors, including primer mismatches and the distance of HIV-1 integration sites to nearby *Alu* sequences [67]. The quantification of integrated HIV-1 DNA has already provided important information on the mechanisms of persistence [4,68,69,71,72,73,74,75,76], and it is frequently used in clinical HIV-1 cure trials [26,77,78,79,80,81]. Its use, in combination with NGS, has been suggested as the most appropriate method for LR quantification [82].

Indeed, whilst measurements of total and integrated HIV-1 DNA are equivalent in ART-suppressed patients, measuring the integrated portion of the viral genome becomes crucial in eradication strategies aiming at reducing the viral reservoir. In this brief report, we present our in-house optimisation method to improve the sensitivity of the standard-free version of the *Alu*-gag PCR assay. We have tested a different primer combination, widely validated to detect different subtypes ensuring wide application of the assay [83], with the aim of detecting an elevated number of integrated HIV-1 DNA molecules.

## 2. Materials and Methods

All experiments were performed in accordance with relevant guidelines and regulations.

### 2.1. Integrated HIV-1 DNA Quantification by qPCR

Integrated HIV-1 DNA was quantified with the *Alu*-gag and *Alu*-5LTR assays using the same genomic DNA aliquots. *Alu*-PCR repetitive sampling was performed as previously described [4,65,67] using the primers listed in Table S1. Briefly, isolated genomic DNA undergoes first PCR (PCR1) amplification targeting both human *Alu* and HIV-1 sequence (run in 40 replicates), allowing for the exponential amplification of integrated HIV-1 DNA. Here, unintegrated HIV-1 DNA forms are amplified in a linear fashion and can be discriminated from integrated amplicons through performing an additional amplification with only the HIV primer being run in parallel (herein referred to as ‘HIV-1 control’; run in 20 replicates per sample and standard). Subsequently, such pre-amplified HIV-1 DNA is quantified using HIV-1 specific qPCR. The mean cycle of quantification (Cq) obtained by the ‘HIV-1 control’ provides the background signal that is used to set the threshold under which a sample can be defined as positive for integration [67]. All PCR1 reactions were run in 20 µL containing 250 nM and 1500 nM of forward and reverse primers, respectively, 1× Promega GoTaq mix and 0.02 U/µL GoTaq polymerase (Promega), 10nM dNTPs (Promega) and DNA extract ranging from 50–250 ng/reaction depending on sample availability. The DNA input was normalised over cellular HIV-1 DNA with the same quantity used for both the *Alu*-gag and *Alu*-5LTR amplifications. The thermocycling conditions used in this assay were previously described by Liszewski [65]. In brief, they comprised 2′ at 95 °C; 40 cycles of the following steps: 95 °C for 15″–50 °C for 15″–70 °C for 3.30′; followed by 15″ at 70 °C. Subsequently, 2 µL of PCR product was used as input for the qPCR containing 400 nM of primers, 200 nM of probe, 1X qPCR SuperMix with ROX (Roche Molecular System) and water to a final volume of 10 µL. qPCR was performed on 2 qPCR machines following validation of the systems (data not shown): Roche qPCR System (supported by Light-Cycler 480 SW 1.5 software, Roche, Basel, Switzerland) and AB 7900HT System (supported by SDS 2.2.1 software, Applied Biosystem). Thermocycling conditions were 2′ at 50 °C and 5′ at 95 °C, followed by 45 cycles at 95 °C for 15″, and 60 °C for 1′. Integrated HIV-1 DNA copy numbers were quantified by inputting the Cq values, obtained from the qPCR into a predesigned computational template [67]. We previously demonstrated that the calculation of the errors based on the Poisson distribution lost power when the input of positive wells was minor compared to that of four *Alu*-HIV-1 positive wells, and thus, that a minimum of four positive wells is required for reliable quantification. In the original calculation sheet, a number of integrations per cell were corrected by 0.1 to account for the limit of quantification of the *Alu*-gag assay, that is, 10% of the total HIV-1 integration events. Here, the results are provided as the number of integrated HIV-1 DNA copies per replicate, which did not include the correction factor. This decision was taken to avoid confusion regarding the correction factor which is a direct reflection of the limit of quantification, and as such, is expected to be different between the two integration assays.

### 2.2. Patients Tested with the Alu-gag and Alu-LTR Assay

For this study, we used PBMC obtained from 20 HIV-1 infected patients undergoing fully suppressive ART that were described previously in reservoir quantification studies [61,73,84,85]. In particular, the studies were approved either by UK Central Ethics Committee or by Ethics Committee of Ghent University Hospital (Reference numbers: B670201317826, B670201525241). All patients provided written informed consent. Patient characteristics are summarised in Supplementary Tables S1 and S2. PMBCs were isolated by density gradient centrifugation and stored at −80 °C or −150 °C prior to genomic DNA isolation, as previously described [73,74].

### 2.3. Standard for Integrated HIV-1 DNA Assay

A previously validated integration standard was provided by Prof Una O’Doherty (Center for Aids Research at the University of Pennsylvania) which carried one single copy of integrated HIV-1 DNA with no traces of unintegrated HIV-1 DNA per single CEM-ss cell [65]. This standard was used to compare the *Alu*-gag and *Alu*-5LTR integrated HIV-1 DNA assays, following in-house measurements of the HIV-copies/µL using ddPCR and qPCR. Furthermore, this standard was diluted at one or two copies per replicate in the DNA of an equivalent of 40,000 uninfected genomes (266 ng of Human Genomic DNA, Sigma, Burlington, MA, USA) to analyse assay sensitivity.

An alternative integration standard was generated in-house utilising latently-infected J-LAT cells. These cells carry a single copy of HIV-1 DNA per cell with no detectable virus replication occurring during passage [86]. To partly compensate for the clonality of the HIV-1 integration sites, different J-LAT clones carrying unique integrations sites were obtained from the NIH AIDS reagent programme: 6.3 (cat#9846), 8.4 (cat#9847), 9.2 (cat#9848), 10.6 (cat#9849), 15.4 (cat#9850). Cells were maintained as indicated by the manufacturer. At passage number 2, 5 × 10^6^ cells were frozen in DMSO as performed with PBMCs. Genomic DNA was isolated using QIAGEN blood extraction kit (QIAGEN) according to manufacturer instructions and total HIV-1 DNA was measured by qPCR [87]. Twenty µL of genomic DNA isolated from each of the different cell clones were merged to generate the “J-LAT standard”. Following total HIV-1 DNA quantification [62,87], we tested three different concentrations of this standard: one, two and five copies diluted in 7500 uninfected cells.

### 2.4. Statistics

Data distribution was assessed through descriptive scatter plots. Bland Altman analysis is the preferred statistical method to investigate agreement and bias between the two quantitative assays. By comparing the mean and standard deviation of each measurement, agreement between the two methods can be investigated. Initial validations of the assumption of constant variance and constant difference between *Alu*-gag and *Alu*-5LTR methods were performed by regressing the absolute values of the residuals of the averages and the differences of the averages, respectively. The power of the linear statistics was 81%. Linear correlation and Bland Altman analyses were performed using the Methcomp package (version 1.22.2) in R, as previously described [88].

## 3. Results

### 3.1. Design an Improved HIV-1 Integration Assay (Alu-5LTR)

**Figure 1:**
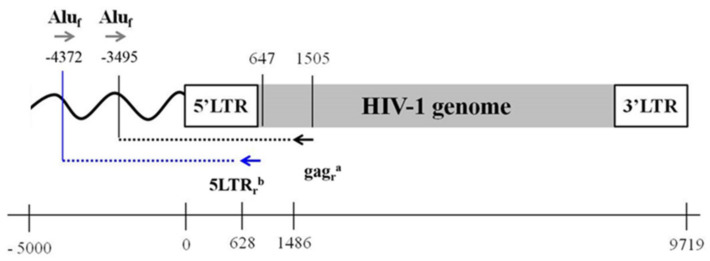
Primer combinations for the PCR1 step in the *Alu*-gag and *Alu*-5LTR assays. Figure depicting the different primers used in the *Alu*-gag and in the *Alu*-5LTR assay for the PCR1 step. ^a^ gag reverse primer that binds HIV-1 genome at 1505–1486 bp; ^b^ 5LTR reverse primer that binds HIV-1 genome at 647–628 bp. The black and the blue dashed lines represent 5 kb long fragments that are the longest that can be amplified by the polymerase used in these assays. With the *Alu*-gag assay integration events that are up to 3495 bp away from the human Alu sequence can be amplified, whereas with the *Alu*-5LTR assay integration events that are up to 4372 bp from an Alu sequence can be amplified. LTR = long terminal repeats, f = forward, r = reverse.

In order to improve the previously used *Alu*-gag standard-free assay, we applied an alternative primer combination to the first PCR (Figure 1) using primers that were previously validated in other approaches employed to measure integrated HIV-1 DNA [61].

In particular, we replaced the HIV-primer (which binds the gag region in the *Alu*-gag assay) with a well-known validated primer that is located at the border between the LTR and splice donor 1 of the HIV-1 sequence, closer to the 5′-end of the HIV-1 genome (647 bp) [83] (Figure 1). Using a Polymerase that amplifies up to 5 kb, we then allowed for amplification of the integrated HIV-1 DNA closer to human Alu sequence, thus improving the number of integration events that could be detected. The choice of the alternative HIV primer was made by cross-validation of the primer sequence using a combined in silico and in vivo evaluation using different HIV-1 DNA primers [61] that allowed for reliable amplification of different HIV-1 subtypes. We established that the efficiency of the second PCR was similar between the validated standard-free *Alu*-gag and the novel proposed assay, as well as being like other validated assays and with little room for improvement. However, in order to avoid overlaps with the primers in the PCR1 in the LTR region, we decided to use the primers described in Avettand-Fènoël 2009 [83]. Meanwhile, we decided to use the same probe of the *Alu*-gag assay in order to run the same patient sample in the same detection plate to ensure higher consistency.

### 3.2. Testing of Alu-5LTR in Patients with Undetectable Viral Load Undergoing Suppressive Antiretroviral Therapy

**Figure 2:**
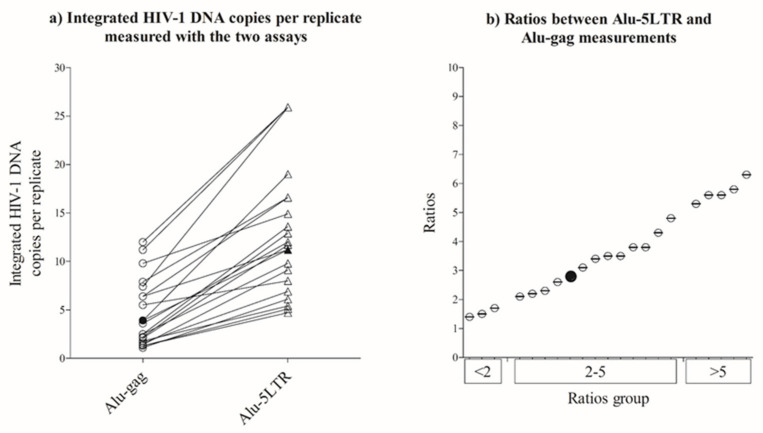
*Alu*-LTR assay showed higher sensitivity in measuring integrated HIV-1 DNA as compared to *Alu*-gag assay. (**a**) integrated HIV-1 DNA copies per replicate measured with *Alu*-gag (black-empty dots) and *Alu*-5LTR (black-empty diamonds) assays. (**b**) representation of ratios between measurements obtained with *Alu*-5LTR and *Alu*-gag assays. Each black-empty dot or triangle represents one patient, the black filled dot or triangles represents the integrated standard. The patients and integrated standard are divided into 3 groups based on the ratios (<2, 2–5 and >5), as shown on *x*-axis.

We then tested the two assays in 20 patients undergoing suppressive ART. We obtained quantification of integrated HIV-1 DNA with both methods in 20/20 samples (100%, Figure 2a). Overall median integrated HIV-1 copies per replicate were 5 (range: 1–12) and 14 (range: 5–26) with the *Alu*-gag and *Alu*-5LTR assays, respectively. The median ratio between the integrated HIV-1 DNA copies measured with *Alu*-5LTR vs *Alu*-gag was 3.5 (range: 1.4–6.3). Most samples (12/20, 60%) had ratios between 2 and 5, with a few being below (3/20, 15%) or above (5/20, 25%) this range (Figure 2b). Additionally, we tested the HIV-1 DNA standard and found that the median ratio between integrated HIV-1 DNA copies measured with the *Alu*-5LTR vs *Alu*-gag was 2.8 (Figure 2b).

### 3.3. Bland Altman Analysis of the Alu-5LTR vs. Alu-gag Assays

**Figure 3:**
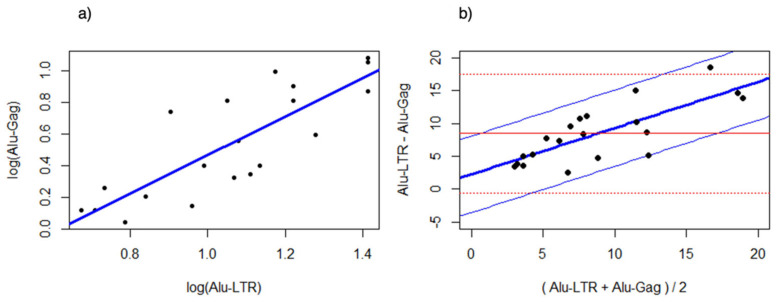
(**a**) Linear regression analysis of the log transformed data from *Alu*-gag and *Alu*-5LTR assays. The trend-line is marked in blue. (**b**) Bland Altman plot of the nontransformed data, showing the difference between both methods (thick blue line) and the 95% confidence intervals (thin blue lines) when constant difference would be assumed. The red lines indicate the differences and the 95% confidence interval in case the difference between both methods would be constant.

Linear correlation of log-transformed data for both assays demonstrated a positive correlation (slope: 1.2125, R^2^ = 0.68, *p* = 8.55 × 10^−6^) (Figure 3a).

Initial assumption testing for the Bland Altman analysis revealed that the variation was constant (*p* = 0.176). However, the assumption for constant difference between the *Alu*-5LTR and *Alu*-gag method was violated. Indeed, a Bland Altman analysis of the nontransformed data showed that the differences between both methods increased when the average estimated value increased (Figure 3b). Hence, the bias between the *Alu*-5LTR and *Alu*-gag was proportional and not constant. Regression analysis of the differences over the average values between *Alu*-5LTR and *Alu*-gag revealed that the *Alu*-5LTR quantified, on average, two times more integrated HIV-1 DNA compared to the *Alu*-gag assay (Figure 3b. *Alu*-LTR = 3.47 + 2.09 *Alu*-gag; standard error at 95% confidence ± 4.49; *p* = 5.48 × 10^−5^).

### 3.4. Testing of a Previously Used HIV-1 DNA Standard to Define the Lower Input Needed to Allow Reliable Poisson Quantification

To define the sensitivity of the assay, we tested different inputs of the ‘integration standard’ and analysed the number of positive wells among the 40 replicates of *Alu*-gag or *Alu*-5LTR. As explained in the methods section, four replicates needed to be scored as “positive for integrated HIV-1 DNA” to achieve reliable Poisson quantification. Indeed, calculation of the errors for each measurement with less than four positive replicates would be unreliable, leading to false-positive or negative results, as discussed previously [67]. In this type of experiment, we aimed to identify which HIV-1 DNA input led to as few as four positive wells being classified as positive.

**Figure 4:**
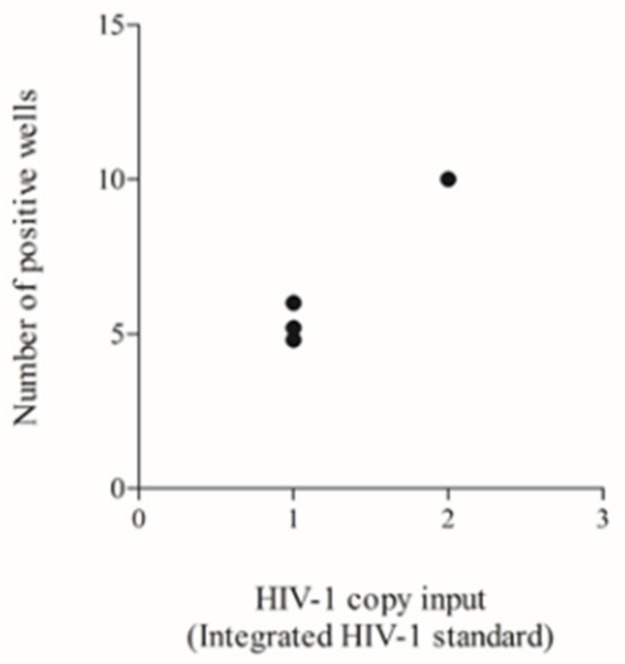
Testing of a previously used HIV-1 DNA standard to define the lower HIV-1 DNA input for reliable Poisson quantification. Number of positive wells obtained by testing one and two copies of integrated HIV-1 standard. Each black dot represents one single run.

We initially tested two HIV-1 copies diluted in the human genome equivalent to 40,000 cells, which is equal to 0.5 proviruses in 10,000 cells and which confirmed the sensitivity of the *Alu*-gag, as previously demonstrated [65]. This input produced 10 positive replicates, which is above the threshold of the four positive reactions required for reliable quantification [67]. Therefore, we reduced the input down to one copy in 40,000 cells, which is equivalent to 0.25 proviruses in 10,000 cells, and repeated the test in three independent runs. In these additional tests, we obtained five and six positive reactions in 2/3 (67%) and 1/3 (33%) experiments, respectively (Figure 4). This number of positive wells was still above the threshold of four positive reactions [67], and thus, this result demonstrated that the *Alu*-LTR assay can reliably detect fewer HIV-1 DNA input copies compared to the *Alu*-gag assay. We did not perform further testing with inputs lower than 0.25 proviruses in 10,000 cells, because we wanted to avoid obtaining unreliable results below the validated threshold (i.e., four positive wells).

### 3.5. Testing of a Cellular Standard to Be Used for the Initial Setting of the Alu-5LTR Assay: Towards Cross-Laboratory Harmonisation

**Figure 5:**
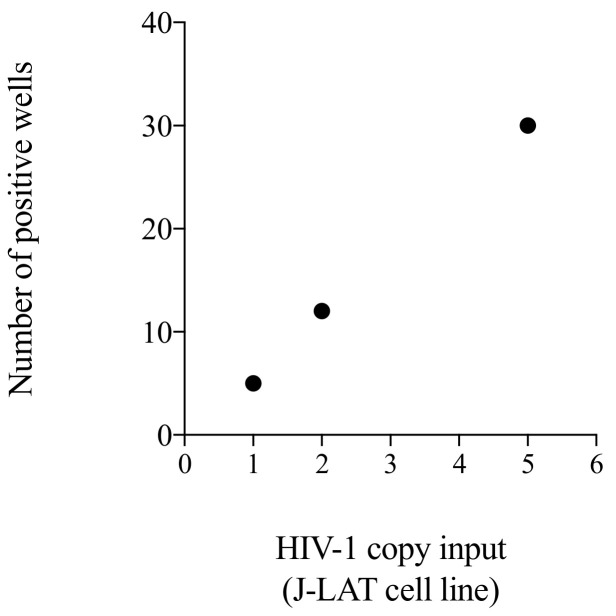
Testing of a standard based on J-LAT cells.

To enable interlaboratory validation of the assay, we generated a calibrator based on a combination of J-LAT cell lines that would allow us to compare assay sensitivity in different laboratory settings. We tested three different HIV-1 DNA inputs: the lowest, the highest and one midpoint sampling. By comparing the number of wells scoring positive for integrated HIV-1 DNA copies, we determined that the number of positive wells and HIV-1 DNA input correlated well through linear regression analysis (Figure 5, R square = 0.99). As expected, we found that the number of positive wells increased with elevated HIV-1 DNA input: one, two and five HIV-1 copy inputs produced 5, 12, and 30 positive wells, respectively.

Number of positive wells obtained by the testing of one, two or five copies of J-LAT standard with the *Alu*-5LTR assay. Each black dot represents one single run. Correlation analysis.

## 4. Discussion

Measuring integrated HIV-1 proviral DNA is essential for the study of reservoir dynamics in eradication studies. However, the qPCR quantification assays currently available are challenging, and any improvements would aid the field in better monitoring the beneficial effects of strategies aimed at HIV-1 cure. Here, we outline the difficulties posed in measuring HIV-1 integration within cells and in monitoring the LR. Further, we present improvements to a PCR-based integrated HIV-1 DNA assay with increased sensitivity to proviral DNA in PBMC. We utilised primer sets binding to a highly conserved region of the HIV-1 genome, closer to the 5′ integration site, in order to improve the efficiency of PCR amplification. Our results suggested that this method was more sensitive in quantifying integrated HIV-1 DNA in patients undergoing effective ART.

Over time, different approaches have been used to quantify integrated HIV-1 DNA levels [65] via qPCR. Of all the methods, the *Alu*-HIV-1 PCR assay demonstrates a good compromise between accuracy and clinical application [89]. One of these assays is the *Alu*-gag, which we improved to be standard-free and to achieve a 10% threshold of detecting integration events [67]. We developed this assay further to improve the sensitivity of the assay changing the HIV-1 primer, binding toward the end of the viral genome. By using the same approach used in the previously published *Alu*-gag assay, we would expect to produce fragments of a similar length but which were more likely to contain both the *Alu* and the HIV-1 sequences, ensuring detection of the integrated HIV-1 DNA. Our results on both patient testing and standards confirmed our assumption. In the majority of tested patients, our *Alu*-5LTR assay improved the quantification of integrated HIV-1 DNA by two to five-fold compared to the *Alu*-gag method. The results on the ‘integration standard’ with the two assays indicated that *Alu*-5LTR provided a two-fold increase in sensitivity. This improved sensitivity also provides a lower limit of detection with the *Alu*-5LTR assay, i.e., 0.25 proviruses in 10,000 genomes, which is two times lower compared to the previously described *Alu*-gag assay.

Our data indicated that the *Alu*-5LTR assay showed increased efficiency of detection in some patients, but not in others. This difference does not seem to be associated with HIV-1 subtype, considering that the majority of patients were infected with subtype B. A Bland Altman analysis revealed that the increased difference between the two assays was correlated linearly with a higher average level of detected integrated HIV-1 DNA. The reason for these differences should be discussed in the context of recent works describing the composition, characterisation and heterogeneity of the HIV-1 genomes in different patients. It has been demonstrated that the majority of HIV-1 proviral reservoir sequences harbour deletions within the HIV-1 genome, including Gag [25,42,90,91]. Thus, it may be possible that LTR-based assays lead to more accurate quantification because they overcome the hurdle of primer mismatch caused by deletion in Gag. Nonetheless, our 5-LTR assay showed superiority in quantification also in the “integrated standard”, which can be presumed to contain intact gag regions. Another aspect that should be discussed is the clonal expansion of HIV-infected cells, as demonstrated by Maldarelli [92]. This clonal expansion may lead to nonrandomly distributed integration sites, and may bias integrated HIV-1 quantification in some patients with high clonality of infected cells. We could speculate that some cellular populations carry conserved integration sites with specific distribution with regard to the distance from an *Alu* sequence. In this case, if the whole cell population has integration sites that are very close to an *Alu* sequence, using the *Alu*-gag or the *Alu*-5LTR should not make a substantial difference on the outcome. In contrast, if the HIV-1 is integrated at a considerable distance from an *Alu* site, the use of the *Alu*-5LTR could represent an improvement. However, since we do not have data on the HIV-1 integration sites of the presently studied patients, we cannot prove this assumption. The debate on the lack of heterogeneity of the integration sites also affects the interpretation of the correction factor used in the calculation of the integrated HIV-1 DNA in cells [65,67,89]. In fact, if it is correct that some patients have HIV-1 integration sites that may facilitate or inhibit the detection of the integrated HIV-1 DNA, it cannot be assumed that the assay performs with the same level of sensitivity for each single patient. Hence, we would not encourage the use of integrated HIV-1 data for absolute quantification to discriminate individual patients, but rather, recommend the use this assay to investigate longitudinal changes within patients or relative changes between larger patient cohorts.

One previous study explored the possibility of targeting the LTR region in PCR1 [63]; this assay has been used in other works [52,93]. The author described a two-step method for the quantification of integrated HIV-1 DNA, where PCR1 was run with a primer binding to one region of the HIV-1 LTR very close to the one we targeted [63]. Of note, in that study, the detection limit was 1.2 proviruses in 10,000 cells. In another study, Vandergeeten et al. [52] demonstrated a quantification of integrated HIV-1 DNA as low as 10 copies/10^6^ cells in 1/31 patients (3.2%); however, the quantification was performed utilising purified CD4 T-cells, which are a demonstrated source of the virus reservoir, and hence, carry many more copies of HIV-1 DNA. Our approach adds to previous methods by demonstrating improved sensitivity in PBMC and not purified CD4-T cells when using an amplification PCR setting that includes one single primer binding the HIV-1 LTR sequence and one single primer binding the *Alu* sequence. Furthermore, we used an innovative quantitative method that avoids the use of a standard due to the use of the Poisson distribution statistics [67], rendering the assay more applicable on a larger scale. However, it must be noted that for a fair and direct comparison with Vandergeeten’s method, the *Alu*-5LTR assay should be performed with purified CD4 T cells, or alternatively, Vandergeeten’s method could be used to test the same PBMC samples reported in this manuscript. Unfortunately, neither were accessible due to a lack of samples.

Despite the standard-free setting of this assay, we acknowledge the fundamental importance of testing an integration standard for a reliable methodology setup in different laboratories. With this in mind, we designed an integrated HIV-1 DNA standard obtained by mixing different clones of the J-LAT cells. This cell line was selected because it harboured numerous integrated HIV-1 DNA copies that did not change over cell passaging [86]. Furthermore, each clone carries HIV provirus in distinct genome locations, expecting different HIV-Alu distances, thus serving as an ideal standard representing the random integration observed in patients. Such a standard is easy to generate and can be used to calibrate the validation of this assay. Nonetheless, we acknowledge that this is only the first step towards the setting up of these novel methods, and that further validations are needed.

This study presents some limitations. First, even if we used widely validated primers known to detect different subtypes [61], primer fidelity is a hurdle when quantifying a highly variant virus such as HIV-1 [65,83]. This should be considered when studying subjects infected with various HIV subtypes. Further, this study did now allow for comparison with QVOA because of sample unavailability. The results obtained using this in vitro assay will provide valuable information, even though it is important to highlight that these methods need to be validated with clinical outcomes such as time to viral rebound in treatment interruption studies, given that QVOA is an imperfect measure.

## 5. Conclusions

Here, we have demonstrated that our primer combination improved the quantification of integrated HIV-1 DNA copies as compared to any previously used methodology using peripheral blood. Additionally, our method has the advantage of avoiding the CD4 T-cell purification step. Furthermore, achieving improved sensitivity lowers the level of patient material that is needed for this assay to be conducted, with consequent benefits for settings with limited sample availability. We applied the Poisson principles for quantification, which simplified the procedure by avoiding having to use a standard that needs to be included in each separate run. Undoubtedly, future modifications will be made to the many assays quantifying the variant virus life-cycle stages and which will be utilised in assaying the effectiveness of strategies aimed at restricting, or indeed, eradicating HIV-1.

## Data Availability

All data available are contained in this manuscript.

## Supplementary Materials

The following are available online at https://www.mdpi.com/article/10.3390/life11121410/s1; Table S1. Primer sequences (5′-3′) used in the integrated HIV-1 DNA assays, Table S2. Patients’ information, Table S3. Patients’ information.
