# Supplementary material for: Measuring Proviral HIV-1 DNA: Hurdles and Improvements to an Assay Monitoring Integration Events Utilising Human Alu Repeat Sequences
Source: Life (Basel). 2021 Dec 16;11(12):1410. doi: 10.3390/life11121410 (PMC8706387; doi:10.3390/life11121410)
Supplement: Supplementary file 1 [file life-11-01410-s001.zip › life-1509693-supplementary-done.pdf]

Supplementary Material

Table S1. Primer sequences (5′-3′) used in the integrated HIV-1 DNA assays. All primers and probes were validated in previous assays (Avettand-Fenoel, Chaix et al. 2009, Liszewski, Yu et al. 2009).

	Target	*Alu*-gag Assay	*Alu*-5LTR Assay
PCR 1	Human *Alu* (sense)	GCCTCCCAAAGTGCTGGGATTACAG	
	HIV-1 (anti-sense)	GTTCCTGCTATGTCACTTCC	GTTCGGGCGCCACTGCTAGA
PCR2	HIV-1 LTR (sense)	TTAAGCCTCAATAAAGCTTGCC	(NEC005) GCCTCAATAAAGCTTGCC
	HIV-1 LTR (anti-sense)	GTTCGGGCGCCACTGCTAGA	(NEC131)GGCGCCACTGCTAG GATTTT
	HIV-1 LTR (probe)	FAM/CCAGAGTCA/ZEN/CACAACAGACGGGCACA/3IABKFQ/	

Table S2. Patients' information

					Pre-ART		On ART		
patient ID	HIV-1 subtype	Age	Gender	ART	CD4 nadir	VL	CD4 count	Total HIV-1 DNA	Log VL
		(years)	(M/F)	(years)	(cells/mm3)	(copies/ml)	(cells/mm3)	(copies/10^6PBMC)	(copies/ml)
1	G	47	M	10.3	97	5.11	369	593.0	<1.3
2	B	37	F	10.3	13	5.47	1032	315.0	<1.3
3	B	41	M	9.4	234	4.99	757	3151.0	<1.3
4	02_AG	43	M	3.8	270	5.29	558	436.0	<1.3
5	B	49	M	5.2	230	4.54	746	436.0	<1.3
6	A	51	F	7.3	147	4.74	1541	130.0	<1.3
7	B	31	M	6.0	350	6.88	612	124.0	<1.3
8	B	45	M	8.0	185	>5.00	647	910.1	<1.3
9	B	38	M	7.0	146	4.82	525	463.1	<1.3
10	B	75	M	6.7	358	4.91	615	279.2	<1.3
11	B	45	M	16.3	46	5.26	708	341.4	<1.3
12	B	43	M	11.2	149	4.38	660	407.5	<1.3
13	B	34	M	6.2	230	4.68	601	621.6	<1.3
14	B	59	M	17.6	201	5.19	1020	349.8	<1.3
15	B	47	M	15.9	0	5.07	871	132.4	<1.3

16	B	62	M	19.1	63	6.17	908	653.9	<1.3
17	B	59	M	19.3	187	4.54	855	291.4	<1.3
18	B	49	M	12.6	44	5.93	275	251.1	<1.3
19	02_AG	53	M	5.9	268	5.04	938	478.8	<1.3
20	01_AE	45	M	5.3	350	3.96	765	205.1	<1.3

Table S3. Patients' information.

Patients' characteristics	
N tot	20
Male N(%)	18 (90%)
Subtype B N (%)	15 (75%)
Subtype G	1 (5%)
Subtype 02_AG	2 (10%)
Subtype 01_AE	1 (5%)
Subtype A	1 (5%)
Age	46 (43-52)
Duration of ART	9 (6-14)
CD4 nadir	186 (89-251)
Pre-ART VL	5 (5-5)
CD4 count sampling	727 (609-890)
Total HIV-1 DNA copies/106 PBMC	346 (272-471)
Plasma HIV-1 RNA > 1.3 log/ml	0
